# Retraction: Does money buy health? evaluation of stock market performance and economic growth in the wake of the COVID-19 pandemic

**DOI:** 10.1371/journal.pone.0311787

**Published:** 2024-10-14

**Authors:** The PLOS ONE Editors

This article [1] was identified as one of a series of submissions for which PLOS has concerns about adherence to journal policies on authorship and systematic manipulation of the publication process.

In addition, the corresponding author stated that the dataset downloaded from the World Development Indicator Database and used in this study is no longer available.

A member of the Editorial Board reviewed the article and data from the version of the World Development Indicator Database available at the time of post-publication follow-up [2]. They noted concerns about the theoretical framework, the study design, and suitability of the reported analyses to address the research question. These issues call into question the validity of the reported results.

The *PLOS ONE* Editors retract this article because of the above concerns. We regret that the issues were not identified prior to the article’s publication.

S agreed with the retraction and apologizes for the issues with the published article. KZ and AK did not agree with the retraction and stand by the article’s findings. AB, IK, and SS either did not respond directly or could not be reached.
